# General practitioners’ attitudes towards and frequency of collaboration with pharmacists in China: a cross-sectional study

**DOI:** 10.1186/s12913-023-10151-0

**Published:** 2023-10-27

**Authors:** Songtao Cai, Xianghui Huang, Connie Van, Wanchao Li, Ming Yan, Yiting Lu, Haixin Li, Zhiling Deng, Panpan Lu, Zhijie Xu

**Affiliations:** 1 Department of General Practice, The Second Affiliated Hospital, School of Medicine, The Chinese University of Hong Kong, Shenzhen & Longgang District People’s Hospital of Shenzhen, Shenzhen, 518172 China; 2 Xinsheng Community Health Service Center, Shenzhen Longgang Central Hospital, Shenzhen, 518116 China; 3 Sydney Pharmacy School, Faculty of Medicine and Health, The University of Sydney, Sydney, NSW 2006 Australia; 4 Lincheng Healthcare Center of Changxing County, Huzhou, 310016 China; 5 Department of General Practice, Sir Run Run Shaw Hospital, Zhejiang University School of Medicine, Hangzhou, 310016 China; 6 Department of General Practice, Tongji University School of Medicine, Shanghai, 200092 China; 7 Department of Pharmacy, The First Affiliated Hospital of Xi’an Jiaotong University, Xi’an, 710061 China; 8 The Eighth Affiliated Hospital, Sun Yat-sen University, Shenzhen, 518033 China; 9 Department of General Practice, Taizhou Municipal Hospital, Taizhou, 318000 China; 10 Department of General Practice, The Second Affiliated Hospital, Zhejiang University School of Medicine, No.88, Jiefang Rd, Hangzhou, Shangcheng District, 310009 China

**Keywords:** Interdisciplinary collaboration, General practitioners, Pharmacists, Cross-sectional study

## Abstract

**Background:**

Building interprofessional working relationships between general practitioners (GPs) and pharmacists is essential to ensure high-quality patient care. However, there is limited Chinese literature on GP–pharmacist collaboration, and few studies have explored GPs’ experiences with pharmacist integration into general practices. This study aimed to investigate GPs’ attitudes towards and frequency of collaboration with pharmacists in China.

**Methods:**

This cross-sectional study used an online self-administered questionnaire integrating two scales, ATCI-GP and FICI-GP, which had been translated and validated to investigate 3,248 GPs from February 15 to March 15, 2023 across Zhejiang Province, China. Descriptive analyses were used, and the factors associated with GPs’ frequency of collaboration with pharmacists were explored using logistic regression analysis.

**Results:**

A total of 2,487 GPs (76.6%) responded and consented to participate in the survey; 52.3% were male and the mean age was 35.4 years. Most GPs agreed that they shared common goals and objectives with pharmacists when caring for patients (90.0%), and pharmacists were open to working with them on patients’ medication management (80.8%). However, half of the GPs did not change or seldom changed the patient’s medication on the pharmacist’s advice (51.4%). Logistic regression analysis showed that GPs who were older and had more years of practice were more likely to agree that pharmacists were willing to collaborate, had common goals for treatment and that they would change the patient’s medication on the advice of the pharmacist. GPs who had regular communication protocols (adjusted odds ratio_1_ [aOR_1_] = 1.88, 95% CI 1.45–2.45; aOR_2_ = 3.33, 95% CI 2.76–4.02), participated in joint continuing education (aOR_1_ = 1.87, 95% CI 1.44–2.43; aOR_2_ = 2.27, 95% CI 1.91–2.70), provided recommendations for medication review (aOR_1_ = 3.01, 95% CI 2.07–4.38; aOR_2_ = 3.50, 95% CI 2.51–4.86), and communicated with pharmacists during resident training (aOR_1_ = 2.15, 95% CI 1.78–2.60; aOR_2_ = 1.38, 95% CI 1.18–1.62) were associated with a more positive attitude towards and higher frequency of cooperation.

**Conclusions:**

GPs in China displayed a positive attitude towards cooperating with pharmacists, but they did not demonstrate a similar level of practice. As environmental determinants impact interdisciplinary collaboration, healthcare managers and policy-makers need to implement measures that foster a supportive environment conducive to interdisciplinary collaboration.

**Supplementary Information:**

The online version contains supplementary material available at 10.1186/s12913-023-10151-0.

## Introduction

Inappropriate medication use has become a major concern of global health, with significant impact on patient safety, public health, and healthcare systems. The World Health Organization (WHO) policy perspectives of medicines in 2002 stated that more than 50% of medications were prescribed, dispensed, or sold inappropriately, which contributed to a higher incidence of adverse drug events (ADEs) [1–3]. ADEs can compromise patient safety, elevate rates of hospital admission, and lead to inflated healthcare costs, undermining the overall efficiency and accessibility of healthcare services [4–6].

The problem of inappropriate medication use is also prevalent and complex in China, predominantly within primary care settings [7]. Our previous studies revealed that Chinese general practitioners (GPs) encountered multifold barriers to optimizing medication use, necessitating GP-pharmacist collaboration in routine practice [8, 9]. Many studies have demonstrated the benefits of such collaboration, thus leading to decreased medication-related problems and enhanced patient clinical outcomes [10–12]. A systematic review indicated that incorporating pharmacists into the healthcare team could yield significant improvements in the management of blood pressure and glycosylated haemoglobin and a reduction in ADEs [13].

Despite the recognized importance of interprofessional collaboration, the current status of GP-pharmacist collaboration in China remains poorly understood, underscoring the need for further research to better comprehend and promote this vital alliance in primary care. While previous studies have investigated the collaborative practices between GPs and pharmacists in various countries [14–16], they have largely overlooked the unique characteristics of China’s healthcare system. Moreover, these studies have primarily focused on the benefits of collaboration but have not delved extensively into understanding GPs’ attitudes towards collaboration with pharmacists. Additionally, the frequency of collaborative practices and the factors that may influence both attitudes and frequency have not been explored in the context of primary care in China.

In this study, we conducted a survey that aims to gain insights into the perspectives of GPs on their collaboration with pharmacists, the frequency of collaboration, and the factors associated with their attitudes and collaboration frequency. The results can provide valuable insights and contribute to potential strategies for enhancing interdisciplinary collaboration in primary care settings.

## Methods

### Study design, population and setting

From February 15 to March 15, 2023, we conducted a cross-sectional study that surveyed licenced GPs via a self-administered online questionnaire. A random sample of 3,248 GPs who registered as members of the Grassroots Health Association of Zhejiang Province were selected and invited to participate in the survey through a short message service (SMS). GPs who were employed at community healthcare centres or township healthcare centres in Zhejiang Province and had a duration of practice of over one year were included. Informed consent was obtained on the first page of the questionnaire. Each respondent was fully informed about the survey, including its investigator, study aim, main content, and rights and obligations. Online survey completion was tracked, but all survey responses were anonymized. Respondents completed the questionnaire voluntarily, could withdraw from the survey before submission and were assured of the confidentiality of their data. No compensation was offered to the participants. Ethical approval was obtained from the Institutional Review Board of the Second Affiliated Hospital of Zhejiang University, and this study was conducted in accordance with the principles outlined in the Declaration of Helsinki [17].

### Instrument

The questionnaire employed in this study integrated two scales, Attitudes Towards Collaboration Instrument for General Practitioners (ATCI-GP) and Frequency of Interprofessional Collaboration Instrument for General Practitioners (FICI-GP), which were developed and validated by Van and colleagues [18, 19]. The items in the questionnaire were divided into three sections. The first section included questions about the GP characteristics (e.g., age, educational background) and environmental determinants (e.g., GP location, remuneration). The second section included questions about GPs’ attitudes towards collaboration with pharmacists, i.e., how GPs perceived their collaboration status and to what extent GPs trusted the competencies of pharmacists (13 items, 5-point Likert scale). The third section included questions about the frequency of GP collaboration with pharmacists, including how often GPs had mutual interactions and jointly managed a patient’s medication with pharmacists in the recent month (10 items, 4-point scale).

### Translation and validation of the instrument

Since the scales of the ATCI-GP and FICI-GP had not been translated into Chinese, we conducted a cross-cultural adaptation study before the formal survey. We followed the guidelines by Beaton et al. in the process of translation and adaptation, which comprised the five steps below [20].

- Forwards translation: Three bilingual experts independently translated the original English versions of the scales into Chinese. Two of the experts had a medical background and were familiar with the concept of interprofessional collaboration between practitioners and pharmacists. The third expert was a professional translator without a medical background and was not informed about the concept of GP-pharmacist collaboration. This approach ensured diverse perspectives in the translation process.
- Synthesis: The research team compared the three translations item by item against the original scales and integrated them into a single Chinese version. Any discrepancies or differences in the translations were discussed among the team members, and consensus was reached to create a harmonized Chinese version that accurately represented the original scales.
- Back translation: The target scales were back translated into English by two translators who had not seen the original scale. The differences between the back translation versions and the original scales were analysed and compared, and the first draft of the simplified Chinese version was created after adjustments were made.
- Expert committee review: A panel of researchers, physicians, and pharmacists reviewed the Chinese versions of the scales for relevance, clarity, and brevity. Their expertise was crucial in ensuring that the translated scales captured the intended meaning and were appropriate for the target population. The feedback and suggestions provided by the expert committee were considered, and revisions were made accordingly to improve the quality of the Chinese versions.
- Pretesting: Pretesting was conducted among 140 participants to ensure the reliability and validity of the prefinal questionnaire. The Cronbach’s α coefficient of the Chinese version of the ATCI-GP was 0.959, Kaiser‒Meyer‒Olkin (KMO) test values were 0.926, and Bartlett’s test of sphericity (BTS) results showed that χ^2^ = 1709.456, P < 0.05. The Cronbach’s α coefficient of the Chinese version of the FICI-GP was 0.965, the KMO test value was 0.933, and the BTS results showed that χ^2^ = 1440.893, P < 0.05, which showed good reliability and validity.

In the final Chinese version of the questionnaire, the original items that made up the ATCI-GP and FICI-GP scales were essentially unchanged; however, we made several modifications to the respondents’ characteristics and environmental determinants: (a) The professional title and educational background of the participating GPs were added; (b) The classification and geographical division of general practices were removed; (c) The options for the distance between the offices of GPs and pharmacists were modified; (d) The response scale of FICI-GP was modified to a four-point scale to evaluate the frequency of collaboration (i.e., “none”, “1–2 times”, “3–4 times”, and “5 or more times”) to mitigate recall bias. The questionnaire was expected to take participants 3–5 minutes to complete.

### Data collection

A self-administered survey was disseminated through Questionnaire Star (Changsha Ranxing Information Technology Co., Ltd), a professional online questionnaire platform in China. Questionnaire Star allows participants to fill out the questionnaires through a mobile phone, tablet or computer, with obvious advantages including speed, ease of use and low cost. The content of the Chinese version of the questionnaire was imported and edited on the Questionnaire Star website (https://www.wjx.cn/), and an online link was generated. Subsequently, we disseminated the online link to the selected GPs by SMS, and they could click on the link to start. The survey required a response for all items; thus, there were no missing data. The data were collected immediately after the participants submitted the completed questionnaires and entered into a web-based database by specialized investigators to ensure accuracy.

The questionnaire could only be submitted 90 s after respondents completed the first item to ensure that they took the time to fully understand each item before responding. Each respondent with a unique IP address could submit the questionnaire only once. A text message reminder was sent to all GPs two weeks after the initial survey to ensure an appropriate response rate. After the survey was closed, we downloaded the questionnaire data from the Questionnaire Star platform and manually checked all data. Invalid questionnaires were removed if they had obvious inconsistency of answers (e.g., a 45-year-old GP with 2 years of practice).

### Statistical analysis

We conducted descriptive analysis to present categorical variables, and GPs’ attitudes towards and frequency of collaboration with pharmacists are reported as numbers and percentages. To explore the associated factors of GPs’ attitudes towards and frequency of collaboration with pharmacists, we chose a priori to focus on several rating-scale statements as the main outcomes of interest because they are the most direct reflection of attitudes and frequency regarding collaboration. The statements “The pharmacist is open to working together with me on patients’ medication management” and “The pharmacist and I share common goals and objectives when caring for the patients” are hereafter referred to as “attitudes towards joint medication management” and “attitudes towards common goals”, respectively. The responses to these two statements were categorized as either agree (including strongly agree and agree) or disagree (including neither agree nor disagree, disagree, or strongly disagree). Logistic regression analysis was conducted to evaluate both unadjusted and adjusted associations between the two main statements and the characteristics of the respondents, as well as environmental determinants.

Similarly, the statements “I adjusted patient medication after the pharmacist’s recommendation” and “I involved the pharmacist in decisions regarding medication management” are hereafter referred to as “adoption of pharmacist’s recommendation” and “pharmacist involvement in medication management”, respectively. The responses of frequency (none, 1–2 times, 3–4 times, and 5 or more times) were then examined using logistic regression to assess unadjusted and adjusted associations between the two main statements and respondents’ characteristics and environmental determinants. We used a two-tailed α value of 0.05 to define statistical significance in all analyses. The Statistical Package for Social Sciences version 26.0 (SPSS Inc., Chicago, IL, USA) was used for all statistical analyses.

## Results

### GP characteristics

A total of 2,487 (76.6%) GPs responded and consented to participate in the survey; slightly more than half (52.3%) were female with a mean age of 33.2, and 47.7% were male with a mean age of 37.8. The GPs had been in practice between 1 and 33 years, with a mean of 11.3 years. More than 90% of GPs had a college or higher education, and 38.7% reported a professional title of attending physician (Table 1).

**Table 1:** Participant characteristics and environmental determinants (n = 2,487)

Characteristic	Data
Sex	
Female	1,185 (47.7)
Male	1,302 (52.3)
Professional title	
Resident	752 (30.2)
Attending physician	963 (38.7)
Vice chief physician	531 (21.4)
Chief physician	241 (9.7)
Education background	
Less than a bachelor’s degree	223 (9.0)
Bachelor’s degree	1,881 (75.6)
More than a bachelor’s degree	383 (15.4)
Age, y	
Mean (SD)	35.4 (7.9)
Range	24–58
Years of practice, y	
Mean (SD)	11.3 (8.7)
Range	1–33
**Environmental determinants**	
Office location	
Next door	119 (4.8)
Same floor	1,864 (75.0)
Different floor	504 (20.2)
Regular communication protocol	
Yes	1,804 (72.5)
Joint continuing education	
Yes	1,623 (65.3)
Agreeing incentive system could influence collaboration	
Yes	1,369 (55.1)
Providing recommendations of medication review	
Yes	2,304 (92.6)
Communication with pharmacists during resident training	
Yes	194 (7.8)

### Environmental determinants

Three-quarters of GPs worked on the same hospital floor as the pharmacy, and 4.8% worked next door to the pharmacy. Most participants (92.6%) reported that they refer their patients for medication review, and 72.5% of GPs regularly communicated with the pharmacist they consulted most often. In the previous year, 65.3% of GPs participated in joint continuing education events or meetings with a pharmacist. Approximately half (55.1%) of the GPs agreed that the availability of remuneration influenced their decision to work with pharmacists in medication management. Only 7.8% of GPs had contact with pharmacists regarding drug therapy during their residencies.

### GPs’ attitudes towards collaboration with pharmacists

**Fig. 1:**
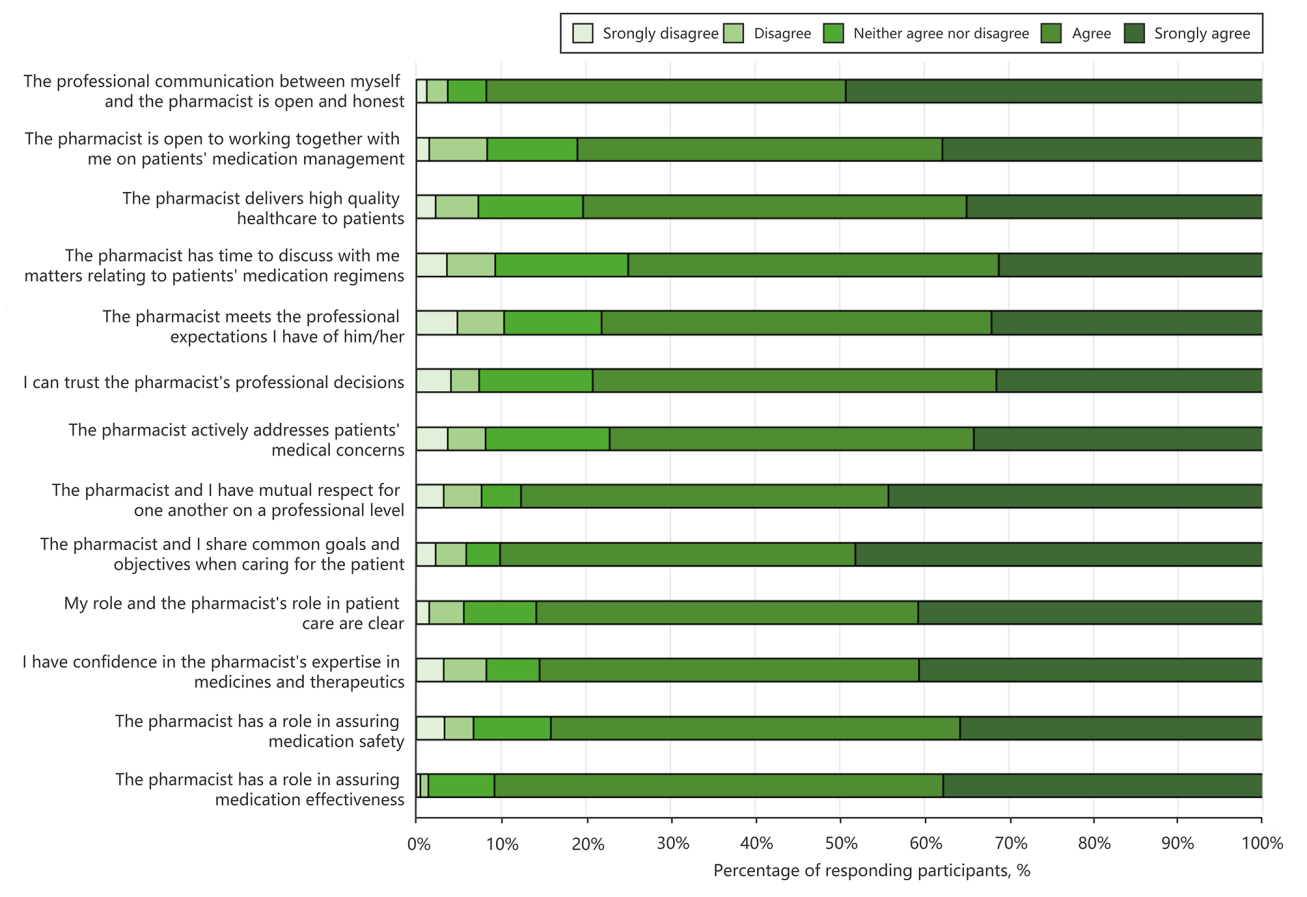
GP attitudes towards their collaboration with pharmacists

Most GPs agreed or strongly agreed that their communication with pharmacists was open and honest (91.7%) and that pharmacists had time to discuss matters related to patients’ medication regimens (74.9%) (Fig. 1). 90% of GPs agreed or strongly agreed that they shared common goals and objectives with pharmacists when caring for the patients and that pharmacists were open to working together with them on patients’ medication management (80.8%). Most GPs trusted pharmacists’ professional decisions (79.0%) and expertise in medication therapy (85.3%) and believed that they delivered high-quality healthcare to patients (80.3%).

### Frequency of GPs’ interprofessional collaboration with pharmacists

**Fig. 2:**
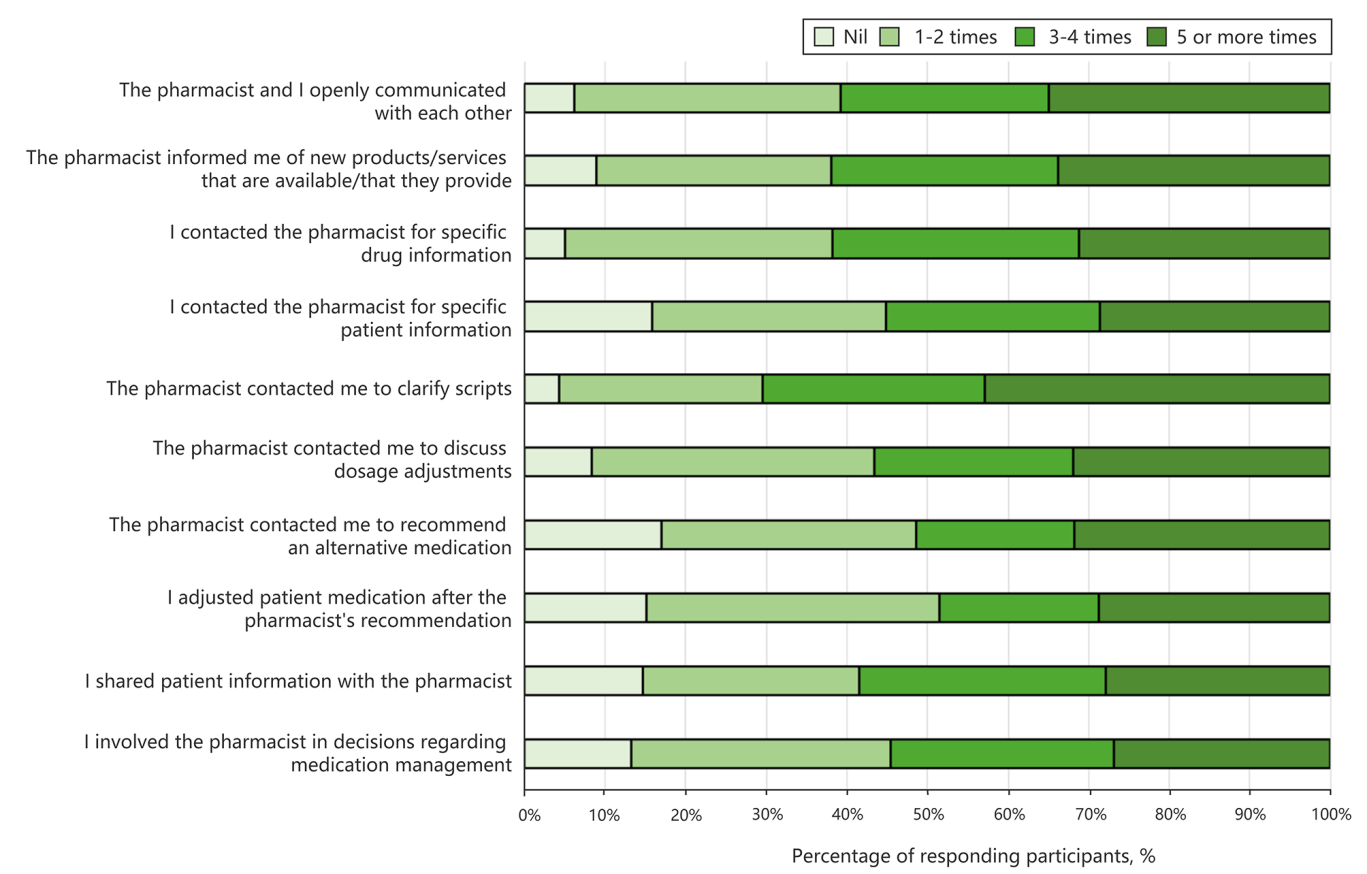
Frequency of GP collaboration with pharmacists

In the previous month, many GPs reported that they did not or seldom (less than twice per month) changed a patient’s medication on the pharmacist’s advice (51.4%) or involved the pharmacist in decisions about medication management (45.4%) (Fig. 2). However, most GPs frequently (more than three times per month) contacted the pharmacist for specific information about a medication (61.7%) and shared patient information with the pharmacist (58.4%). GPs also reported that pharmacists frequently (more than three times per month) contacted them to confirm the contents of a prescription (70.4%), discuss dosage adjustments (56.6%), and recommend substitute medications (51.3%).

### Factors associated with GPs’ attitudes towards and the frequency of collaboration

The logistic regression analysis found no statistically significant differences in attitudes towards joint medication management (adjusted odds ratio [aOR], 1.13; 95% CI, 0.93–1.39) and common goals (aOR, 0.89; 95% CI, 0.67–1.19) between male and female physicians. GPs’ professional titles, educational background, and years of practice did not exert a significant influence on their attitudes towards collaboration with pharmacists. However, GP age and a few environmental determinants (i.e., joint continuing education, having regular communication protocols, receiving recommendations for medication review, and communication with pharmacists during resident training) were associated with GP perceptions of pharmacist willingness to collaborate and their shared treatment goals with pharmacists (Table 2).

**Table 2:** Logistic regression analysis of GP attitudes towards collaboration with pharmacists

	Attitudes towards joint medication management, OR (95% CI)		Attitudes towards common goals, OR (95% CI)	
**GP characteristics**	Unadjusted	Adjusted^a^	Unadjusted	Adjusted^a^
Sex				
Female	1 [Reference]	1 [Reference]	1 [Reference]	1 [Reference]
Male	1.20 (0.97–1.48)	1.13 (0.93–1.39)	0.85 (0.65–1.10)	0.89 (0.67–1.19)
Professional title				
Resident	1 [Reference]	1 [Reference]	1 [Reference]	1 [Reference]
Attending physician	0.91 (0.61–1.37)	0.93 (0.62–1.41)	0.97 (0.57–1.64)	0.96 (0.56–1.62)
Vice chief physician	1.13 (0.87–1.49)	1.21 (0.92–1.61)	1.15 (0.98–1.34)	1.07 (0.91–1.27)
Chief physician	0.80 (0.63–1.01)	0.90 (0.70–1.15)	1.21 (1.02–1.44)^b^	1.19 (0.99–1.41)
Education background				
Less than a bachelor’s degree	1 [Reference]	1 [Reference]	1 [Reference]	1 [Reference]
Bachelor’s degree	0.65 (0.39–1.09)	0.89 (0.42–1.36)	0.91 (0.75–1.09)	0.93 (0.79–1.12)
More than a bachelor’s degree	0.84 (0.53–1.34)	0.90 (0.68–1.21)	0.96 (0.76–1.21)	0.94 (0.80–1.13)
Age, y	1.00 (0.98–1.03)	1.04 (1.02–1.07)^b^	1.02 (1.00-1.04)^b^	1.10 (1.06–1.14)^b^
Years of practice, y	1.01 (0.99–1.03)	1.00 (0.98–1.02)	1.00 (0.99–1.01)	0.98 (0.95–1.01)
**Environmental determinants**				
Office location				
Next door	1 [Reference]	1 [Reference]	1 [Reference]	1 [Reference]
Same floor	1.32 (1.07–1.64)^b^	1.09 (0.88–1.37)	1.05 (0.78–1.43)	1.09 (0.80–1.50)
Different floor	0.70 (0.51–0.98)	0.75 (0.53–1.07)	0.70 (0.51–0.94)^b^	0.74 (0.53–1.03)
Regular communication protocol	1.80 (1.40–2.33)^b^	1.88 (1.45–2.45)^b^	1.82 (1.34–2.49)^b^	1.89 (1.36–2.63)^b^
Joint continuing education	1.77 (1.39–2.26)^b^	1.87 (1.44–2.43)^b^	1.67 (1.23–2.25)^b^	1.58 (1.13–2.21)^b^
Agreeing incentive system could influence collaboration	1.04 (0.82–1.33)	1.01 (0.79–1.30)	0.83 (0.61–1.13)	0.93 (0.67–1.29)
Providing recommendations of medication review	2.62 (1.84–3.74)^b^	3.01 (2.07–4.38)^b^	2.82 (1.27–6.27)^b^	2.73 (1.21–6.13)^b^
Communication with pharmacists during resident training	2.04 (1.69–2.45)^b^	2.15 (1.78–2.60)^b^	2.46 (1.08–5.60)^b^	2.22 (0.98–5.01)

^a^ Adjusted for age, sex, professional title, years of practice, and educational background

^b^ Significant association (P < 0.05)

GPs with a greater number of years of practice showed an increased likelihood of adopting a pharmacist’s recommendation (aOR, 1.06; 95% CI, 1.04–1.08), and older GPs were found to be more inclined to involve the pharmacist in decisions about medication management (aOR, 1.07; 95% CI, 1.05–1.09). In contrast, the results indicated that male physicians tended to decline collaboration with pharmacists in both instances (aOR_1_, 0.77; 95% CI, 0.66–0.90; aOR_2_, 0.75; 95% CI, 0.64–0.88). There were no statistically significant differences observed in the frequency of GP-pharmacist collaboration based on professional titles and educational background. Additionally, the results indicated a notable trend where the frequency of collaboration tended to decrease as the distance of the office location increased. GPs who received recommendations for medication reviews from pharmacists, had a regular communication protocol, participated in joint continuing education, and had communication with pharmacists during resident training were found to collaborate with pharmacists more frequently (Table 3).

**Table 3:** Logistic regression analysis of GPs’ frequency of collaboration with pharmacists

	Adoption of pharmacist’s recommendation, OR (95% CI)		Pharmacist involvement in medication management, OR (95% CI)	
Characteristic	Unadjusted	Adjusted^a^	Unadjusted	Adjusted^a^
Sex				
Female	1 [Reference]	1 [Reference]	1 [Reference]	1 [Reference]
Male	0.96 (0.84–1.12)	0.77 (0.66–0.90)^b^	0.73 (0.63–0.84)^b^	0.75 (0.64–0.88)^b^
Professional title				
Resident	1 [Reference]	1 [Reference]	1 [Reference]	1 [Reference]
Attending physician	1.19 (0.98–1.44)	1.21 (0.98–1.49)	0.93 (0.79–1.14)	0.96 (0.78–1.19)
Vice chief physician	0.88 (0.70–1.09)	0.79 (0.61–1.01)	0.92 (0.73–1.16)	1.02 (0.79–1.32)
Chief physician	0.99 (0.74–1.32)	0.72 (0.50–1.03)	1.17 (0.87–1.57)	1.44 (0.99–2.08)
Education background				
Less than a bachelor’s degree	1 [Reference]	1 [Reference]	1 [Reference]	1 [Reference]
Bachelor’s degree	0.78 (0.58–1.05)	0.92 (0.66–1.27)	1.12 (0.86–1.47)	1.19 (0.84–1.68)
More than a bachelor’s degree	1.03 (0.92–1.15)	1.07 (0.96–1.21)	0.91 (0.67–1.26)	0.86 (0.58–1.28)
Age, y	0.99 (0.98-1.00)	1.00 (0.98–1.02)	1.02 (1.01–1.03)^b^	1.07 (1.05–1.09)^b^
Years of practice, y	1.01 (1.00-1.02)^b^	1.06 (1.04–1.08)^b^	1.01 (1.00-1.02)^b^	0.99 (0.99–1.01)
**Environmental determinants**				
Office location				
Next door	1 [Reference]	1 [Reference]	1 [Reference]	1 [Reference]
Same floor	0.71 (0.49–1.04)	0.67 (0.46–0.98)^b^	0.80 (0.67–0.97)^b^	0.75 (0.62–0.90)^b^
Different floor	0.56 (0.40–0.81)^b^	0.49 (0.34–0.71)^b^	0.87 (0.61–1.22)	0.68 (0.47–0.97)^b^
Regular communication protocol	3.10 (2.59–3.71)^b^	3.33 (2.76–4.02)^b^	4.04 (3.33–4.90)^b^	4.90 (4.04–5.96)^b^
Joint continuing education	2.28 (1.94–2.69)^b^	2.27 (1.91–2.70)^b^	1.96 (1.66–2.31)^b^	1.88 (1.58–2.24)^b^
Agreeing incentive system could influence collaboration	0.87 (0.67–1.14)	0.94 (0.71–1.24)	1.03 (0.78–1.37)	1.06 (0.81–1.41)
Providing recommendations of medication review	3.25 (2.37–4.47)^b^	3.50 (2.51–4.86)^b^	2.31 (1.69–3.17)^b^	2.59 (1.87–3.58)^b^
Communication with pharmacists during resident training	1.47 (1.27–1.72)^b^	1.38 (1.18–1.62)^b^	1.17 (1.01–1.37)^b^	1.26 (1.07–1.48)^b^

^a^ Adjusted for age, sex, professional title, years of practice, and educational background

^b^ Significant association (P < 0.05)

## Discussion

In this cross-sectional analysis, we conducted an online questionnaire to explore GPs’ attitudes towards and frequency of collaboration with pharmacists, as well as the factors influencing such collaborations in Zhejiang Province, China. The study revealed that most respondents had a favourable attitude towards collaboration with pharmacists; however, the frequency of actual collaborations was still insufficient. We also identified several environmental determinants that exhibited a significant association with attitudes towards and the frequency of interprofessional collaboration.

In the first part of our survey, we assessed the attitudes of GPs towards collaboration with pharmacists in primary care settings. Our findings suggest that nearly 90% of GPs perceive their communication with pharmacists as open and honest, and more than 80% exhibited trust in the pharmacists’ expertise and willingness to cooperate on matters of medication management. These findings align with those reported in the study by Ameerah et al., suggesting that GPs exhibit similar attitudes towards cooperation with pharmacists, particularly regarding communication, collaboration willingness, and recognition of pharmacists’ competencies [21]. Nevertheless, our study found that approximately one-quarter of GPs felt that pharmacists lacked sufficient time to discuss patients’ medication regimens. This echoes previous research, which highlighted the lack of time as a potential barrier to achieving effective interdisciplinary collaboration in primary care settings [22, 23]. Another barrier to effective collaboration arises from issues related to limited office space and a lack of open channels for communication, which may lead to insufficient information exchange between GPs and pharmacists. A qualitative investigation executed in Australia highlighted the viewpoint of GPs that direct, face-to-face interaction could facilitate enhanced mutual collaboration; however, physical constraints within general practices and time pressures often curtail this communicative interaction [24]. The role of primary care pharmacists is frequently confined to that of “dispensers”, as most of their working hours are allocated to medication dispensing and consequently they have less time for dynamic interaction with GPs. This pattern suggests a structural problem in which pharmacists’ potential for broader clinical roles is underutilized due to routine and spatial constraints [25, 26].

We also investigated the frequency of interaction between GPs and pharmacists during routine clinical practice. We found that more than half of the GPs routinely reached out to pharmacists for specific drug-related information, while pharmacists initiated contact with GPs to clarify prescription details. These findings align with results from studies conducted in both Australia and the United Kingdom [19, 21]. Despite these routine interactions, a noteworthy finding from our study was that nearly 50% of GPs rarely adjusted patient medications based on a pharmacist’s recommendation and that they seldom involved pharmacists in medication management. This observation presents a potential gap in the collaborative practices between these two core health professions and calls for a deeper examination into the reasons for this behaviour among GPs. One possible reason is that GPs may not be fully aware of the functions of pharmacists and the potential role they could play in primary care [27]. While there is compelling evidence demonstrating that the integration of pharmacists into primary care teams can enhance patient outcomes and satisfaction, the benefits remain underappreciated among many GPs. For instance, a New Zealand survey highlighted that nearly 60% of GPs viewed the primary role of community pharmacists as being restricted to dispensing medication, yet a mere 17% acknowledged the potential for community pharmacists to assist doctors in crafting medication management plans [28]. This perception suggests a significant underestimation of the scope of a pharmacist’s professional capabilities and thus limits pharmacist involvement in primary care and reduces opportunities for interdisciplinary teamwork. A lack of confidence may also originate from the pharmacists in the form of self-doubt. Prior research indicates that approximately one-third of GPs reported that pharmacists only sometimes had the confidence to make clinical decisions [21]. Pharmacists’ lack of confidence might stem from a variety of factors, such as insufficient clinical training, a lack of exposure to certain clinical scenarios, or even the traditionally defined role of pharmacists that focuses on dispensing rather than decision-making in patient care [21]. This perception in turn leads to a reluctance from GPs to engage pharmacists in the clinical decision-making process and medication management.

The study examined the factors associated with GPs’ attitudes towards and frequency of collaboration with pharmacists. The findings revealed that there were no statistically significant differences in attitudes towards collaboration between male and female physicians in terms of joint medication management and common treatment goals, which is consistent with previous research on gender-based differences in interprofessional collaboration [29]. However, it is noteworthy that male physicians tended to decline collaboration with pharmacists, highlighting the importance of exploring the underlying reasons for this gender disparity. Possible influences could include gender-related factors such as differences in communication styles or professional norms. Furthermore, professional titles and educational background did not exert a significant influence on GPs’ attitudes towards and frequency of collaboration. These findings suggest that factors related to professional qualifications may not strongly determine GPs’ attitudes towards and engagement in collaboration. However, GPs with more years of practice and older physicians exhibited a higher likelihood of adopting pharmacist recommendations and involving them in medication management decisions. This observation suggests that as GPs accumulate experience, their recognition of the value and expertise that pharmacists bring to collaborative work increases, leading to a greater willingness to engage and cooperate with pharmacists.

The logistic regression analysis revealed that various environmental determinants, such as the presence of a regular communication protocol, engagement in joint continuing education, pharmacist provision of medication review recommendations, and communication with pharmacists during resident training, were significantly associated with more positive attitudes and increased frequency of collaboration. The regular communication protocol serves as a critical platform to foster interaction between GPs and pharmacists. It aids in cultivating mutual understanding, establishing robust communication, and fostering cooperative relationships. As exemplified in a UK study, over 90% of GPs reported that pharmacists’ activities were orchestrated through mutual agreements between the two professional groups [21]. This finding underscores the necessity of instituting formal regular protocols or meetings in primary care settings. Previous studies also revealed the direct impact of medication review recommendations as well as engagement in joint continuing education on GPs’ attitudes and collaborative frequencies [18, 30–32]. Communication with pharmacists during residency training presents a pivotal opportunity to inculcate an understanding of the potential contributions pharmacists can make to medication management during the formative years of a physician’s career. The timely establishment of this understanding is essential, as professional identity perceptions are often solidified early in a physician’s career [33–35]. Thus, integrating early exposure to and interaction with pharmacists in the training of future physicians could be instrumental in fostering better collaborative attitudes and practices.

Moreover, our study revealed a positive correlation between geographical proximity and collaboration frequency between GPs and pharmacists in primary care settings. It is conceivable that closer physical locations provide more opportunities for regular interaction, allowing for the development of rapport and fostering positive working relationships. This observation is corroborated by numerous previous studies [18, 21, 36, 37]. For instance, research conducted by Harding et al. indicated that GPs who worked near on-site health centre pharmacists demonstrated more favourable attitudes towards GP-pharmacist collaboration compared to their counterparts who worked in settings separate from pharmacists [36]. Similarly, a study featuring interviews with GPs in New Zealand showed a widely held belief among practitioners that closer proximity to pharmacists would enhance communication, thereby improving collaboration [37]. These findings emphasize the impact of spatial configurations in healthcare settings on collaborative practice and the potential benefits of rethinking and redesigning physical spaces in healthcare facilities to encourage increased interaction and collaboration between GPs and pharmacists.

Our research highlights several important implications. First, there is a pressing need to fortify the recognition of pharmacists’ roles and augment the active communication between pharmacists and GPs. Achieving this may necessitate a dedicated campaign to enlighten GPs about the broadened scope of pharmacists’ roles, which go well beyond the traditional dispensary functions, highlighting their capacity to contribute significantly to patient care outcomes. Educational tools for this campaign could include structured information sessions, collaborative workshops, or even interprofessional education programs, all with the aim of fostering an atmosphere of mutual respect and collaboration. Second, GPs need an environment that promotes frequent, meaningful interactions with pharmacists in primary care settings. Such an environment is instrumental in ensuring that both parties are thoroughly informed about a patient’s medication management plan. Consequently, this would enable them to work in unison, efficiently ensuring optimal patient outcomes. Future research should therefore focus on identifying effective strategies to create such an environment. In this context, establishing regular, structured communication protocols could be a stepping stone towards facilitating mutual understanding and cooperation. Third, the disparity we observed between positive attitudes and actual collaboration frequency among GPs and pharmacists underscores the role of policy intervention. Healthcare policymakers need to devise supportive policies that facilitate collaboration, potentially by providing shared training programs or interprofessional networking opportunities. Other systemic changes, such as creating integrated care models or redefining job descriptions, could also help overcome barriers to collaboration. Policies promoting the integration of pharmacists into primary care teams could be particularly impactful. They could validate and reinforce the value of the pharmacists’ role in the eyes of other healthcare professionals and patients, which could lead to enhanced patient satisfaction and outcomes.

Our study had several limitations. First, the frequency of collaboration between GPs and pharmacists was assessed using the FICI-GP instrument, which queries GPs about their interactions with the pharmacists they most frequently engage with in the preceding month [19]. While this scale has been translated and validated formally in the Chinese context, it could potentially introduce recall bias, as it relies on participants’ ability to accurately remember and report their interactions [38]. Second, our study sample is limited to a single province in China, and thus, the findings might not be generalizable to other geographical regions. Despite the potential limitations in external validity, the results of our study contribute meaningfully to the understanding of GP–pharmacist collaboration in primary care settings within the sampled region and can provide a reference for future comparative studies in different regions. Third, our sampling methodology may have potentially introduced nonresponse bias. However, we stress that given the robust sample size and the systematic approach to data collection, the impact of nonresponse bias on our conclusions is likely to be minimal.

## Conclusion

While GPs in China display a very positive attitude towards cooperating with pharmacists in primary care settings, the actual frequency of such collaborations needs to increase. Notably, the limited involvement of primary care pharmacists in routine medication management signifies an underrecognition of their potential value. Environmental determinants are essential to contribute to the interdisciplinary collaboration between GPs and pharmacists, underlining the need for healthcare managers and policymakers to implement measures to engender a supportive environment conducive to this collaboration.

### Electronic supplementary material

Below is the link to the electronic supplementary material.

STROBE 2007 (v4) Statement?Checklist of items that should be included in reports of *cross-sectional studies*

## Data Availability

The datasets generated and/or analysed during the current study are not publicly available due to protection of the participants’ anonymity but are available from the corresponding author on reasonable request.

## Abbreviations

GPs: General practitioners
ADEs: Adverse drug events
ATCI-GP: Attitudes towards collaboration instrument for general practitioners
FICI-GP: Frequency of interprofessional collaboration instrument for general practitioners
